# Pomegranate peel supplementation improves performance and lipid metabolism in laying hens

**DOI:** 10.5194/aab-68-629-2025

**Published:** 2025-10-28

**Authors:** Şaziye Canan Bölükbaşı, Hilal Ürüşan

**Affiliations:** 1 Department of Animal Science, Faculty of Agriculture, Atatürk University, Erzurum, Türkiye; 2 Department of Plant and Animal Production, Vocational School of Technical Sciences, Atatürk University, Erzurum, Türkiye

## Abstract

This study investigated the effects of dietary supplementation with pomegranate peel at various levels (0, 50, 100, and 150 mg kg^−1^) in high-energy diets on the performance, egg quality, serum lipid profile, liver enzyme activity, and liver fat ratio of laying hens. A total of 120 Lohmann LSL laying hens, aged 70 weeks, were used in the study. The hens were divided into five experimental groups, each comprising six subgroups. The control group was fed a basal diet, while the treatment groups received high-energy (HE) diets supplemented with 0, 50, 100, or 150 mg kg^−1^ pomegranate peel.

During the 9-week trial, feed and water were provided ad libitum. Results indicated that supplementation with 150 mg kg^−1^ pomegranate peel in the HE diet significantly improved egg production and the feed conversion ratio (FCR; p<0.05 ). Additionally, pomegranate peel supplementation had a positive effect on shell-breaking strength and yolk color. Triglyceride (TG) and very-low-density lipoprotein (VLDL) cholesterol concentrations were significantly reduced in the pomegranate peel supplemented groups compared to the HE + 0 mg kg^−1^ P group.

Dietary supplementation with 100 and 150 mg kg^−1^ pomegranate peel significantly ( P<0.01 ) reduced the liver fat ratio compared with the group fed the high-energy diet alone. Plasma malondialdehyde (MDA) and non-esterified fatty acid (NEFA) levels increased at 50 and 100 mg kg^−1^ dietary pomegranate peel.

In conclusion, it was found that the addition of pomegranate peel to a high-energy diet improved some performance and egg quality criteria and reduced the liver fat ratio. However, more detailed studies are required.

## 1 Introduction

Fatty liver syndrome (FLS) is a significant metabolic disorder in poultry, characterized by excessive fat deposition in the liver. This condition arises from various factors, including genetics, dietary composition, environmental influences, and management practices. High-energy diets, with a large proportion of energy derived from carbohydrates and insufficient protein or lecithin, are known to contribute to the development of FLS (Liu et al., 2016). It adversely impacts poultry production by reducing growth rates, lowering feed efficiency, and increasing mortality, resulting in considerable economic losses. To address these challenges, incorporating lipotropic agents into poultry diets is a promising strategy to reduce liver fat accumulation and improve overall productivity.

Pomegranates (*Punica granatum* L.) have attracted substantial scientific interest due to their high antioxidant capacity and abundance of bioactive compounds, including punicic acid, ellagitannins, and ellagic acid (Al-Salhie et al., 2017). Among its various parts, pomegranate peel is particularly rich in phenolics, flavonoids, tannins, and organic acids, which exhibit hepatoprotective, anti-inflammatory, and anti-diabetic properties (Boussaa et al., 2020; Li et al., 2006; Melgarejo-Sánchez et al., 2021). These compounds play a critical role in mitigating oxidative stress and enhancing liver health, making pomegranate peel a potential functional feed ingredient (Bölükbaşı et al., 2023). Furthermore, pomegranate peel has been traditionally employed in managing metabolic disorders and improving overall health across diverse animal models.

Ellagic acid, a prominent polyphenol in pomegranate flowers, has been extensively studied for its hepatoprotective effects (Long et al., 2019). Its mechanisms of action include the reduction of oxidative stress and the regulation of lipid metabolism, positioning it as a potential therapeutic agent for alleviating FLS in poultry (Pagare et al., 2015). However, while the health benefits of pomegranate fruits and seeds are well documented, the specific applications of pomegranate peel in poultry nutrition remain underexplored, necessitating further investigation.

This study aims to evaluate the effects of dietary supplementation with pomegranate (*Punica granatum* L.) peel on key parameters in laying hens, including performance metrics, egg quality, liver health, serum lipid profiles, antioxidant enzyme activities, and liver fat content. By elucidating the functional roles of pomegranate peel, this research seeks to provide evidence-based dietary strategies to enhance poultry health and productivity while addressing the economic losses associated with FLS.

## 2 Methods

### 2.1 Establishment of trial groups and trial plan

Before initiating the study, ethical approval was obtained from the Atatürk University Veterinary Faculty (no. 2018/01). The study was conducted using 120 Lohmann LSL hybrid laying hens aged 70 weeks. The hens were randomly allocated into five experimental groups, each consisting of 24 birds with six replicates per group (four birds per replicate).

The experimental design included a control group receiving a basal diet (2650 kcal kg^−1^; Table 1) and a high-energy (HE) group fed a diet containing 2850 kcal kg^−1^. The remaining three groups were fed HE diets supplemented with graded levels of pomegranate peel (mg kg^−1^), as specified in Table 1. The feeding trial was carried out over a period of 9 weeks. Feed and water were provided ad libitum throughout the experimental period. During the trial, 16 h of light and 8 h of darkness were applied. The house temperature was adjusted between 18 and 20 °C, and the ventilation conditions were optimized. The antioxidant capacity (DPPH %) of pomegranate peel used in the experiment was determined as 89 %.

**Table 1:** Ingredients (%) and chemical analyses of the basal diet.

Ingredients	Basal	High-energy
	diet	diet
Corn	62.2	63.5
Soybean meal 44–46	17.36	13.15
Corn gluten 60	8.48	10.64
Limestone	9.68	7.65
DCP 18	1.44	1.44
Soy oil	–	2.7
Vitamin-mineral premix^∗^	0.25	0.25
Common salt	0.22	0.33
Sodium bicarbonate	0.16	0.16
L-lysine HCL	0.11	0.10
DL-methionine	0.10	0.08
Analyzed chemical composition (%)		
Dry matter	88.41	88.54
Crude protein	17.52	17.20
Crude fat	2.20	4.84
Crude ash	11.87	10.35
Crude fiber	2.78	2.57
DL-methionine	0.38	0.38
Methionine	0.40	0.41
Lysine	0.76	0.70
ME kcal kg^−1^	2650	2850

^∗^ The premix provided per kilogram of diet: 12 000 IU vitamin A, 2500 IU cholecalciferol (vitamin D3), 30 IU α -tocopheryl acetate (vitamin E), 4 mg menadione sodium (vitamin K3), 3 mg thiamine mononitrate (vitamin B1), 6 mg (riboflavin) vitamin B2, 30 mg niacin (vitamin B3), 10 mg calcium D-pantothenate (vitamin B5), 5 mg pyridoxine (vitamin B6), 0.015 mg cyanocobalamin (vitamin B12), 1 mg folic acid; 0.050 mg D-biotin (vitamin H), 50 mg ascorbic acid (vitamin C), 300 mg choline chloride, 80 mg manganese oxide, 60 mg iron, 60 mg zinc, 5 mg copper, 0.5 mg cobalt, 2 mg iodine, and 0.15 mg selenium.

**Table 2:** Phenolic compound amount of pomegranate peel used in the experiment.

Phenolic	Quantification of
compound	polyphenols (mg kg^−1^)
Ellagic acid	34
Punicalin	8
Gallic acid	12
Punicalagin A	274
Punicalagin B	497

### 2.2 Performance and egg quality criteria

Daily feed intake (subgroup average) and egg weights were recorded biweekly. Feed efficiency was calculated by dividing the amount of feed consumed by the total egg production (kg). Egg yields were recorded daily and expressed as a percentage divided by the number of animals in the subgroup. A total of 12 eggs were taken every 2 weeks from the treatment groups, and egg white, yolk, shell ratio, shell thickness, and Haugh unit were determined.

### 2.3 Blood parameter analysis

At the end of the experiment, blood samples were collected from one animal per subgroup ( n=6 ) via venipuncture and placed into heparinized tubes. The samples were then centrifuged at 3000 rpm for 10 min, and the serum was stored at -80 °C until analysis. Superoxide dismutase (SOD) activity (Sun et al., 1988), glutathione (GSH) level (Tietze, 1969), malondialdehyde (MDA) level (Yoshioka et al., 1979), glutathione peroxidase (GPx) activity (Matkovics et al., 1988), catalase (CAT) activity (Goth, 1991), total protein (TP) levels (Lowry et al., 1951), and non-esterified fatty acid (NEFA) levels in plasma (Biont Chicken NEFA ELISA Kit, cat. no. YLA0179CH) were measured using a BioTek ELISA reader (BioTek Quant MQX200 ELISA reader, USA). TP levels were used to calculate SOD and GPx activity. Plasma cholesterol, glucose, low-density lipoprotein (LDL), high-density lipoprotein (HDL), very-low-density lipoprotein (VLDL), aspartate aminotransferase (AST), alanine aminotransferase (ALT), and triglyceride (TG) values were analyzed in a specialized laboratory.

### 2.4 Liver weights and determination of total lipid in liver tissue

At the end of the experiment, six randomly selected animals from each group were slaughtered, and their liver wet weights were recorded. The livers were then dried at 105 °C for 24 h, and the dry weights were determined. The ratio of wet to dry liver weight was calculated. To quantify the total lipid content in the liver, the Bligh and Dyer (1959) method was employed. Approximately 1 g of homogenized liver tissue was mixed with a solvent mixture of chloroform, methanol, and water ( 2:1:0.9 v/v/v ), and the contents were thoroughly mixed. After phase separation, the chloroform phase, containing the lipids, was collected and washed twice with water to remove residual methanol and polar compounds. The solvent was then evaporated using a rotary evaporator, and the lipid extract was dried under nitrogen. Finally, the dried lipid extract was weighed to determine the total lipid content, which was expressed as a percentage of the liver's original weight. This method reliably quantifies the lipid content in liver tissue, providing insights into the effects of dietary treatments.

### 2.5 Determination of antioxidant capacity and phenolic compounds in pomegranate peel

The antioxidant capacity of the pomegranate peel used in the experiment was determined by the DPPH (2,2 diphenyl-1-picryhydrazyl) method according to the method described by Brand-Williams et al. (1995), and the phenolic compound content was determined quantitatively by high-performance liquid chromatography (HPLC) according to Ávila-Reyes et al. (2018).

#### Statistical analyzes

A Kolmogorov–Smirnov normality test was used to determine whether the data were suitable for normal distribution, and Levene's statistics were used to test the homogeneity of variances. After obtaining the data in accordance with the normal distribution and homogeneous variances, an analysis of variance (one-way ANOVA) was applied, and the differences between the means were determined by a Duncan multiple comparison test. The significance was tested at a 0.05 level, and the SPSS 23.0 package program was used in all analyses (SPSS 23; IBM Corp., 2015).

## 3 Results

### 3.1 Performance

The results showed that the addition of pomegranate peel to the high-energy diet of laying hens did not lead to significant differences in feed intake (Table 3). However, egg weight was significantly decreased in the group fed the high-energy diet without pomegranate peel. The highest ( P<0.05 ) egg production (88.71 %) and the best feed conversion efficiency ( P<0.05 ) were observed in the HE + 150 mg kg^−1^ group (Table 3).

**Table 3:** Effects of different levels of dietary pomegranate peel on the performance of laying hens.

Groups	Feed consumption (g)	Egg weight (g)	Egg production (%)	Feed conversion ratio (g : g)
Control	126.52	62.93^a^	83.83^a,b^	2.40^b^
HE + 0 mg kg^−1^ P	122.95	60.69^b^	78.90^b^	2.61^a^
HE + 50 mg kg^−1^ P	129.55	64.50^a^	87.48^a,b^	2.30^b^
HE + 100 mg kg^−1^ P	120.80	63.60^a^	74.33^c^	2.68^a^
HE + 150 mg kg^−1^ P	128.10	64.19^a^	88.71^a^	2.25^c^
SE	1.41	0.33	1.42	0.05
P	0.315	0.001	0.008	0.025
CI	123–128	62.52–63.87	80.46–86.18	2.33–2.53

^a,b,c^ Means in columns with different superscripts differ significantly at P<0.05 . HE: high-energy diet, SE: standard error, CI: confidence interval for mean, N=6 replicate cages totaling 24 hens per treatment.

### 3.2 Egg quality criteria

The effect of pomegranate peel on some egg quality criteria is shown in Table 4. No significant difference was found between the groups for albumin ratio, yolk ratio, shell ratio, shell thickness, and Haugh unit (Table 4). The addition of pomegranate peel to the diets of laying hens improved shell-breakage strength and yolk color. The difference between the groups in terms of egg shape index was significant ( P<0.01 ), and the lowest value was recorded in the HE + 0 mg kg^−1^ P group.

**Table 4:** Influence of dietary pomegranate peel on egg quality.

Groups	Albumen	Yolk	Shell	Haugh	Shell-breaking	Shell thickness	Egg shape	Yolk color
	ratio (%)	ratio (%)	ratio (%)	units	strength (kg cm^3^)	( µm )	index	score
Control	59.75	29.72	10.52	83.15	0.766^b^	0.436	75.16^a^	9.50^b^
HE + 0 mg kg^−1^ P	57.92	31.37	10.70	81.58	0.223^c^	0.370	69.83^b^	9.50^b^
HE + 50 mg kg^−1^ P	59.12	30.82	10.05	80.41	2.280^a^	0.469	73.16^a^	11.50^a^
HE + 100 mg kg^−1^ P	57.89	31.22	10.88	82.81	2.000^a^	0.421	75.50^a^	10.66^a^
HE + 150 mg kg^−1^ P	59.89	29.14	10.96	80.73	1.968^a^	0.397	73.75^a^	12.00^a^
SE	0.61	0.53	0.18	0.824	0.20	0.013	0.583	0.297
P	0.764	0.645	0.589	0.806	0.000	0.215	0.008	0.009
CI	57.66–60.16	29.35–31.55	10.23–11.01	80.05–83.42	1.03–1.86	0.390–0.447	72.28–74.67	10.02–11.24

^a,b,c^ Means in columns with different superscripts differ significantly at P<0.05 . HE: high-energy diet, SE: standard error, CI: confidence interval for mean, N=6 replicate cages totaling 24 hens per treatment.

### 3.3 Blood serum constituents

When serum biochemical parameters were examined, no statistically significant differences were observed among the groups in terms of ALP, AST, ALT, LDL, HDL, and total cholesterol levels. However, triglyceride and VLDL levels were found to be lower in the groups supplemented with pomegranate peel compared to the HE + 0 group. In addition, a tendency toward reduced glucose levels was observed in the HE + 50 and 100 mg kg^−1^ groups (Table 5).

**Table 5:** Effects of dietary supplementation with different levels of pomegranate peel on serum biochemical parameters of laying hens.

Groups	ALP	AST	ALT	Trigliserit	Glucose	Total cholesterol	LDL	HDL	VLDL
	(U L^−1^)	(U L^−1^)	(U L^−1^)	(mg dL^−1^)	(mg dL^−1^)	(mg dL^−1^)	(mg dL^−1^)	(mg dL^−1^)	(mg dL^−1^)
Control	253.33	244.66	2.00	257.66^d^	260.67^a,b^	114.66	85.33	37.66	38.33^c^
HE + 0 mg kg^−1^ P	259.33	265.66	5.33	1567.00^a^	248.00^b^	193.33	158.00	42.66	313.33^a^
HE + 50 mg kg^−1^ P	415.33	132.66	3.33	793.33^c^	199.00^c^	109.66	85.00	29.33	232.33^b^
HE + 100 mg kg^−1^ P	219.99	170.33	3.66	1105.33^b^	202.33^c^	97.00	143.00	24.00	221.33^b^
HE + 150 mg kg^−1^ P	386.60	264.66	3.33	570.33^c,d^	300.00^a^	91.00	63.00	33.00	114.33^b^
SE	51.43	0.20	0.52	130.51	12.62	18.15	14.40	3.53	29.32
P	0.737	0.098	0.430	0.000	0.025	0.424	0.143	0.560	0.003
CI	196–416	172–258	2.40–4.65	578–1138	214–269	82.2–160	75.97–137.76	25.74–40.91	121–246

^a,b,c,d^ Means in columns with different superscripts differ significantly at P<0.05 . HE: high-energy diet, SE: standard error, CI: confidence interval for mean, N=6 replicate cages totaling 12 hens per treatment.

Table 6 shows the effects of pomegranate supplementation on serum antioxidant enzymes (MDA, GSH, SOD, CAT, GPx) and NEFA. The highest MDA and lowest GSH levels were observed in the group supplemented with 100 mg kg^−1^ pomegranate peel ( P<0.01 ). The lowest SOD, CAT, and GPx levels and the highest NEFA levels were again observed in the HE + 100 mg kg^−1^ P group (Table 6).

**Table 6:** Effects of dietary supplementation pomegranate peel on blood serum biochemical parameters of laying hens.

Groups	MDA	GSH	SOD	CAT	GPx	NEFA
	(nmol L^−1^)	(nmol L^−1^)	(U L^−1^)	(KU L^−1^)	(U L^−1^)	(ng L^−1^)
Control	7.79^c^	2.33^a^	57.16^a^	149.87^a,b^	1.44^b^	0.224^c^
HE + 0 mg kg^−1^ P	7.56^c^	2.41^a^	59.12^a^	154.54^a,b^	1.50^b^	0.216^c^
HE + 50 mg kg^−1^ P	16.13^b^	1.78^c^	51.68^b^	121.57^c^	1.42^c^	0.317^b^
HE + 100 mg kg^−1^ P	31.67^a^	1.36^d^	46.20^c^	106.36^d^	1.10^d^	0.804^a^
HE + 150 mg kg^−1^ P	7.91^c^	2.14^b^	57.89^a^	160.96^a^	1.53^a^	0.208^c^
SEM	2.48	0.10	1.42	5.58	0.041	0.006
P	0.000	0.000	0.001	0.000	0.000	0.000
CI	8.87–19.55	1.78–2.25	51.40–57.50	125–149	1.31–1.49	0.22–0.48

^a,b,c,d^ Means in columns with different superscripts differ significantly at P<0.05 . HE: high-energy diet, SE: standard error, CI: confidence interval for mean, N=6 replicate cages totaling six hens per treatment, MDA: malondialdehyde, GSH: glutathione, SOD: superoxide dismutase, CAT: catalase, GPx: glutathione peroxidase.

### 3.4 Weight and fat ratio of liver

It was found that wet and dry liver weights were significantly higher in the HE + 0 mg kg^−1^ and HE + 100 mg kg^−1^ groups than in the other groups. It was observed that liver fat content was higher in the groups fed the high-energy diet, but the addition of 100 and 150 mg kg^−1^ pomegranate peel to the diet reduced the fat content (Table 7).

**Table 7:** Effects of treatment on hepatic variables of laying hens.

Groups	Liver wet weight (g)	Liver dry weight (g)	Liver fat (%)
Control	20.32^b^	6.05^c^	23.65^b^
HE + 0 mg kg^−1^ P	39.13^a^	14.12^a^	50.06^a^
HE + 50 mg kg^−1^ P	24.85^b^	9.35^b,c^	51.57^a^
HE + 100 mg kg^−1^ P	38.56^a^	12.62^a,b^	26.41^b^
HE + 150 mg kg^−1^ P	24.72^b^	7.48^c^	18.71^b^
SE	2.52	1.017	4.29
P	0.005	0.010	0.004
CI	23.44–34.71	7.43–11.97	24.66–43.10

^a,b,c^ Means in columns with different superscripts differ significantly at P<0.05 . HE: high-energy diet, SE: standard error, CI: confidence interval for mean N=6 replicate cages totaling six hens per treatment.

## 4 Discussion

### 4.1 Productive performance

Pomegranates have been identified as a potential source of valuable nutrients and antioxidants for livestock nutrition (Emami et al., 2015). In the present study, the inclusion of pomegranates in the diet significantly influenced several performance parameters, such as egg production, egg weight, and feed conversion ratio (FCR). While the weight of the eggs was found to be quite low in the group fed a high-energy diet without added pomegranate peel, it was determined that the weight of the eggs increased in the group fed a high-energy diet with added pomegranate peel compared to the former group. This increase in egg production is likely due to the bioactive compounds in pomegranates, such as polyphenols and antioxidants, which are known to enhance metabolic activity, reduce oxidative stress, and improve immune function, ultimately supporting better reproductive performance. These findings are consistent with the results of Saki et al. (2014), who observed a 5 % increase in egg production in laying hens when pomegranates were included in their diet. Similarly, Hosseini-Vashan and Ghaznavi (2018) and Kostogrys et al. (2017) reported improvements in both FCR and egg production when pomegranates were supplemented in laying hen diets, further supporting the positive impact of pomegranates on laying performance. Additionally, Abbas et al. (2017) found improvements in both egg production and feed intake in Japanese quails fed pomegranate peel powder, suggesting that these benefits are not limited to laying hens but may extend to other poultry species. The antimicrobial properties of pomegranates (Howell and D'Souza, 2013) could also contribute to improved health and performance by reducing pathogen-related stress, leading to better nutrient absorption and overall reproductive efficiency.

Furthermore, recent varietal screening of pomegranate peel extracts has demonstrated that the high contents of polar phenols, flavonoids, and condensed tannins (including punicalagin, flavonoid fractions, and chlorogenic acid) contribute significantly to the antimicrobial and antibiofilm activities of the peel against key poultry pathogens such as *Staphylococcus aureus* and *Listeria monocytogenes*, with biofilm inhibition exceeding 70 % in certain varieties (Salim et al., 2023). These findings underscore the fact that pomegranate peel contains a complex phytochemical profile that not only offers antioxidant benefits but may also enhance poultry health by mitigating pathogenic bacteria colonization and biofilm formation in the gastrointestinal tract.

Interestingly, despite the positive effects of pomegranate supplementation on egg production, there was no significant impact on feed intake in our study. This is consistent with the findings of Rajani et al. (2011) and Saki et al. (2014), both of whom reported no significant changes in feed consumption when hens were fed pomegranate-enriched diets.

However, the current study also revealed some negative effects of pomegranate supplementation on egg quality characteristics in the high-energy diet group. Specifically, egg weight decreased in the high-energy group, which contrasts with the findings of Saki et al. (2014), who observed a positive effect of pomegranate supplementation on egg weight in laying hens. Moreover, Saki et al. (2019) found that egg weight increased by 8 % in laying hens fed pomegranate by-products (PBPs), compared to the other treatment groups. This discrepancy between our findings and those of previous studies may suggest that the impact of pomegranate supplementation on egg weight is influenced by various factors, including the specific dietary context, the form of pomegranate used, and the energy content of the diet. Further research is needed to explore these factors and their potential interactions more comprehensively.

Similar to pomegranates, other fruit by-products rich in polyphenols have been investigated for their potential antioxidant and performance-enhancing effects in poultry nutrition. For instance, the by-products of fruit processing, such as grape pomace and grape seeds, have been reported in various studies to exert antioxidant effects in broiler chickens due to their polyphenol and flavonoid content. In the studies conducted by Goñi et al. (2007) and Brenes et al. (2008), dietary inclusion of increasing levels of grape pomace was associated with reduced lipid oxidation in broiler meat and enhanced total antioxidant capacity. However, Surai (2014) has highlighted certain methodological limitations in these studies, pointing out, for instance, that vitamin E in the form of tocopheryl acetate does not possess direct antioxidant activity and that polyphenols were not detected in tissue samples. These issues raise questions regarding the interpretation of the observed effects. Furthermore, the low bioavailability of polyphenols and the lack of detailed understanding of their mechanisms of action suggest that conclusions about their physiological efficacy should be approached with caution.

### 4.2 Egg quality criteria

Eggshell quality is a critical factor in poultry production, influencing both the marketability and storage stability of eggs. The strength, color, and thickness of eggshells are affected by a range of factors, including breed, age, health status, and nutrition (Ahmadi and Rahimi, 2011). Among these factors, eggshell thickness and specific gravity have been widely recognized as important indicators of eggshell strength, which is a key determinant of the egg's ability to withstand handling, storage, and transport (Wells, 1967). Furthermore, poor eggshell strength leads to significant economic losses, as eggs with weakened shells are more prone to breakage, resulting in higher rates of wastage during transportation and retail (Caner and Yüceer, 2015). In the current study, it was shown that the addition of pomegranate peel to the diet increased the eggshell-breaking strength in laying hens. This result suggests that pomegranate supplementation may improve the mechanical properties of eggshells, potentially enhancing their durability and reducing breakage rates. However, it should be considered that this effect may not be equally effective in all situations, and the supplementation may vary depending on different species and dietary combinations. Additionally, while the observed benefits are promising, further research is needed to investigate the potential side effects of long-term and high-dose supplementation, particularly in understanding its impact on overall poultry health and performance.

The observed increase in eggshell strength could be attributed to the rich antioxidant content of pomegranates, particularly polyphenols and flavonoids, which are known to support calcium metabolism in laying hens. Enhanced calcium utilization leads to stronger, more resilient eggshells. Furthermore, pomegranates may help to bolster immune function and reduce inflammation, contributing to overall better health and, subsequently, improved eggshell quality.

The addition of pomegranates to the diet had no significant effect on albumen ratio, yolk ratio, shell ratio, Haugh unit, or shell thickness in this study. Similarly, numerous studies have shown that dietary supplementation with pomegranate by-products does not affect specific gravity or eggshell thickness, which serve as indirect markers of eggshell-breaking strength in laying hens and quails (Ghahtan et al., 2019; Sharma et al., 2020). Some researchers, such as Saki et al. (2014) and Çetingül et al. (2019), also reported that the supplementation of pomegranate molasses did not positively impact egg quality parameters like eggshell thickness, Haugh unit, eggshell weight, or eggshell-breaking strength.

Egg yolk color is an important factor influencing consumer preferences. In European countries, consumers tend to prefer eggs with darker yolk colors (Hernandez et al., 2005). In our study, pomegranate peel positively affected egg yolk color, likely due to the presence of pomegranate pigments. The primary pigments responsible for the color of pomegranates are anthocyanins (Ben-Simhon et al., 2015). Anthocyanins are bioactive compounds widely found in higher plants. In addition to giving color to plants, they possess strong antioxidant properties (Noda et al., 2002; Moga et al., 2021) and exhibit bacteriostatic activity (Naz et al., 2007). Anthocyanins are also regarded as safe and effective food colorants (Manach et al., 2004). However, since anthocyanins are water soluble, carotenoids (which are lipophilic molecules) tend to deposit more efficiently in egg yolks than anthocyanins. Consequently, numerous studies have observed an increase in yolk color when pomegranate is added to the diet of laying hens (Ishikawa et al., 1999; Kostogrys et al., 2017).

Eggs come in various shapes, which can be differentiated using a shape index (SI). The most common shapes are sharp, normal (standard), and round eggs, with SI values of <72 , 72–76, and >76 , respectively (Sarıca and Ersayın, 2004). The shape index values (%) were significantly affected by all treatments, ranging from 69.83 % to 75.50 %. The group fed with a high-energy diet had a shape index of 69.83. Eid et al. (2021) reported that feeding hens with 2 % and 4 % pomegranate peel powder diets did not significantly affect the shape index. Normal chicken eggs typically have an elliptical shape. Unusually shaped eggs, such as long and narrow, round, or flat-sided eggs, are not classified as Grade-AA or Grade-A eggs (USDA, 2000). Round and unusually shaped eggs have a poor appearance, do not fit properly in preformed packaging, and are more prone to rupture during shipping compared to normal-shaped eggs (Jacob et al., 2000).

### 4.3 Blood serum constituents

Pomegranates are rich in bioactive components, including ellagic acid, ellagitannins (such as punicalagins), punicic acid, flavonoids, anthocyanidins, anthocyanins, estrogenic flavonols, and flavones (Gil et al., 2000). These compounds are known for their antioxidant, anti-inflammatory, and potential therapeutic effects, particularly in the context of metabolic health. Previous studies have shown that pomegranates can reduce cholesterol absorption and increase cholesterol excretion in hyperlipidemic type 2 diabetes patients, leading to significant beneficial effects on cholesterol metabolism enzymes (Yılmaz and Usta, 2010).

The present study also revealed that serum triglyceride (TG) and very-low-density lipoprotein (VLDL) concentrations were higher in the high-energy (HE) groups compared to other treatment groups. This finding is consistent with the well-documented observation that diabetic patients often have issues with cholesterol packaging and tend to exhibit elevated serum TG levels.

In previous animal studies, such as those conducted by Bagri et al. (2009), the oral administration of pomegranate flower aqueous extract significantly reduced fibrinogen, total cholesterol (TC), triglycerides (TGs), low-density lipoprotein cholesterol (LDL-C), and tissue lipid peroxidation, while simultaneously increasing high-density lipoprotein cholesterol (HDL-C) and glutathione content in streptozotocin-induced diabetic rats. These results suggest that pomegranate supplementation may have potential benefits for managing dyslipidemia and oxidative stress in diabetic conditions. Furthermore, in vivo and in vitro studies have consistently shown that pomegranate juice, peel, and fruit extracts possess antioxidant and anti-inflammatory activities, positively impacting glycemia, insulin levels, dyslipidemia, blood pressure, and foam cell formation (Jurenka, 2008; Rana et al., 2010).

In our study, serum triglyceride and VLDL levels were lower in the groups supplemented with pomegranate peel than in the group fed only a high-energy diet. This suggests that pomegranate supplementation may contribute to improving lipid metabolism and reducing lipid accumulation in the bloodstream. Saki et al. (2019) also observed a decrease in plasma cholesterol and an increase in plasma HDL levels when pomegranate by-products (PBPs) were included in the diet of laying hens. Similarly, Eid et al. (2021) reported that adding 2 % and 4 % pomegranate peel powder to the laying hen diet resulted in a reduction in serum triglyceride and LDL concentrations. These findings support the idea that pomegranate supplementation may have beneficial effects on lipid profiles in poultry, which could be attributed to the high content of flavonoids and tannins in pomegranate by-products.

The hypolipidemic effects of pomegranate peel observed in this study are likely mediated through multiple complementary mechanisms. Bioactive compounds, including punicalagins and ellagic acid, may inhibit pancreatic lipase activity, leading to a reduction in intestinal fat absorption and, consequently, lower circulating triglyceride levels. Additionally, the antioxidant components of pomegranate may reduce the oxidative modification of LDL particles, which is a critical step in the development of atherosclerosis. Aviram et al. (2000) demonstrated that pomegranate juice decreased the susceptibility of LDL to oxidation by enhancing serum paraoxonase activity and reducing lipid peroxidation, which ultimately lowered foam cell formation and the size of atherosclerotic lesions in experimental models. Together, these mechanisms may help to explain the improved lipid profile observed in the present study.

The lipid-lowering effects of pomegranates are likely due to the combined action of their antioxidant properties and their high fiber content. Pomegranates' fiber binds with bile salts in the intestinal tract, reducing intestinal transit time and increasing the excretion of sterols, which in turn affect cholesterol metabolism. As a result, this process may lead to lower serum cholesterol levels through increased bile secretion and the reduction of cholesterol absorption (Jiménez-Moreno et al., 2009; Mateos et al., 2012). Therefore, the positive effects of pomegranate supplementation on lipid metabolism observed in this study could be attributed to its complex bioactive composition, which promotes healthier lipid profiles and overall metabolic health.

In the current study, the addition of 150 mg kg^−1^ pomegranate peel to the diet resulted in a significant increase in antioxidant enzymes (GPx and CAT). The excessive release of free radicals during stress conditions reduces the activity of antioxidant enzymes such as GPx, SOD, and CAT, which play crucial roles in detoxifying free radicals and maintaining cellular health. These findings align with those of Eid et al. (2021), who reported that the inclusion of pomegranate peels in the diets of laying hens significantly improved GPx, SOD, and CAT activities. Similarly, Saha and Ghosh (2009) found that punicic acid, a key component of pomegranate, increased the activity of catalase, glutathione, and SOD in rats. The observed effects of pomegranate flower, peel, and extracts on plasma oxidation indices may be attributed to the role of phenolic compounds in inhibiting oxidative processes, as previously highlighted by Surai et al. (2019). In recent years, evidence has emerged that oxidative stress plays a crucial role in the development and perpetuation of inflammation (Lauridsen, 2019). For instance, in pigs, dietary supplementation with grape seed and grape marc meal extracts or hop extract (10 g kg^−1^ diet) has been shown to reduce the expression of several proinflammatory genes in different intestinal segments (duodenum, ileum, colon), while also decreasing the abundance of some potentially pathogenic bacteria (Fiesel et al., 2014). These findings suggest that polyphenols exert not only anti-inflammatory but also antimicrobial effects, which together may contribute to improved intestinal health.

However, the current study also observed that the MDA (malondialdehyde) value was significantly higher ( P<0.01 ) in the 50 and 100 mg kg^−1^ pomegranate groups. This increase in plasma MDA levels may be indicative of enhanced oxidative stress, a point that deserves closer consideration. Numerous studies to date have proven the antioxidant effects of pomegranate peel and pomegranate seed oil, and their positive effects on MDA levels are well documented. However, some studies on the subject have also reported increased MDA levels. For instance, Bölükbaşı et al. (2023) found that adding 1 or 1.5 mL kg^−1^ of pomegranate seed oil to laying hen diets increased MDA levels. Similarly, Saki et al. (2014) found that adding 5 % pomegranate seed meal to laying hen diets had no effect on total antioxidant capacity in the serum but did increase MDA levels. The researchers suggested that the high PUFA content of pomegranate seed oil may increase plasma MDA levels because PUFAs are more susceptible to oxidation. Pomegranate peel is also known to have a high PUFA content (Akhtar et al., 2015). Conversely, a meta-analysis of numerous human studies examined the relationship between the duration of pomegranate consumption and MDA levels. Lorzadeh et al. (2022) reported that MDA levels decreased with administration for more than 8 weeks, while no significant effect was observed with shorter administrations.

Similarly, in the present study, the elevated MDA values, particularly at higher supplementation levels, could be linked to the oxidative vulnerability of the fatty acid composition in pomegranate peel. Although the pomegranate is generally recognized for its antioxidant properties, the fatty acid profile potentially rich in PUFAs might render it prone to oxidation under physiological conditions, especially when administered in higher doses. This paradoxical effect underlines the complex interaction between antioxidant and pro-oxidant mechanisms in the biological system, warranting a more nuanced understanding of dose-dependent responses.

In addition to MDA, plasma non-esterified fatty acids (NEFAs), also known as free fatty acids (FFAs), were also elevated, which may further support the presence of oxidative and metabolic stress. Elevated NEFA levels are traditionally associated with adverse metabolic outcomes, including insulin resistance, as increased adiposity was thought to drive excessive NEFA release into circulation (Karpe et al., 2011). However, more recent studies suggest that NEFA release per unit of adipose tissue may actually decrease with increased fat mass, complicating the interpretation of elevated NEFA levels in this context.

Nevertheless, the concurrent increase in both MDA and NEFA levels observed in the present study raises the possibility of an imbalance in redox homeostasis, potentially driven by the interaction of bioactive components in pomegranate peel with lipid metabolism. This highlights the need for further studies to delineate the specific pathways through which pomegranate peel affects oxidative stress and lipid mobilization in laying hens.

### 4.4 Weight and fat ratio of liver

It was determined that using high-energy feed had a significant effect ( P<0.05 ) on the wet weight and dry weight of the liver. The lowest wet and dry liver weights were observed in the control, HE + 50 mg kg^−1^ P, and HE + 150 mg kg^−1^ P groups. Moreover, a significant difference ( P<0.01 ) was identified between the groups concerning liver fat ratio based on dry matter. The groups HE + 0 mg kg^−1^ P and HE + 50 mg kg^−1^ P exhibited the highest fat ratios.

Ivy and Nesheim (1973) reported that the liver fat ratio could exceed 40 % of dry weight and even reach up to 70 % in cases of fatty liver. In this study, the liver fat ratio in the group fed with high-energy feed (HE + 0 mg kg^−1^ P) was found to be 47.24 % higher compared to the control group. Consistent with these findings, several studies have also reported that high-energy feed increases the liver fat ratio in layer hens raised in cage systems (Akkılıç and Tanyolaç, 1975; Jensen et al., 1970).

The groups supplemented with 50 and 100 mg kg^−1^ of pomegranate peel had significantly lower liver fat content than the group without pomegranate peel. This may be due to the antihyperlipidemic properties of pomegranate peel. It is thought that the phenolic compounds and antioxidants found in pomegranate peel reduce liver fat accumulation by regulating lipid metabolism. Similar results have been reported in the literature, with the antihyperlipidemic effect of pomegranate peel being clearly demonstrated in a study conducted on rats by El-Hadary and Ramadan (2019). These data suggest that pomegranate peel provides antioxidant capacity and positively influences lipid metabolism, thereby reducing liver fat content.

Additionally, pomegranate extract has been reported to reduce liver weight and fat ratio in Zucker fatty (ZF) rats (El-Rashedy et al., 2011). While limited, molecular studies on pomegranates' effects on fatty liver have provided valuable insights. For example, Xu et al. (2009) investigated the efficacy of pomegranate fruit extract on the liver of Zucker diabetic fatty rats and human hepatic (HepG2) cells. Their findings demonstrated that pomegranate fruit extract supplementation decreased liver weight, triglyceride (TG) content, and lipid droplet formation. The mechanism involves stimulating the activation of gene expression related to lipid metabolism, including PPAR α , carnitine palmitoyltransferase-1 (CPT1), and acyl-CoA oxidase (ACO), while reducing the expression of stearoyl-CoA desaturase.

Furthermore, pomegranate extract has shown improvement in hyperlipidemia, hyperglycemia, and fatty heart conditions in diabetic Zucker mice (Huang et al., 2005a, b). These results highlight the potential therapeutic effects of pomegranate extract on fatty liver and metabolic disorders. 

## 5 Conclusion

As a result, it was determined that the addition of 150 pomegranates to the high-energy diet had a positive effect on FCR and egg production, increased shell-breaking strength, and yolk color values, and significantly decreased serum TG and VLDL values supplemented with pomegranate groups. As a result, it was concluded that the addition of pomegranate to the feeds was successful in eliminating the diseases that may occur due to lipid metabolism in laying hens and in improving the performance values. In addition, this study will be a source for future studies on fatty liver disease.

## Data Availability

The data presented in this study are available free of charge for any user upon reasonable request from the corresponding author.

## References

[bib1.bib1] Abbas RJ, Al-Salhie KCK, Al-Hummod SKM (2017). The effect of using different levels of pomegranate (Punica granatum) peel powder on productive and physiological performance of Japanese quail (Coturnix coturnix japonica). Livest Res Rural Dev.

[bib1.bib2] Ahmadi F, Rahimi F (2011). Factors affecting quality and quantity of egg production in laying hens: A review. World Appl Sci J.

[bib1.bib3] Akhtar S, Ismail T, Fraternale D, Sestili P (2015). Pomegranate peel and peel extracts: Chemistry and food features. Food Chemistry.

[bib1.bib4] Akkılıç M, Tanyolaç A (1975). Kafeste beslenen tavuk rasyonlarındaki enerji düzeyinin karaciğer yağlanmasıüzerine etkisi. AÜ Vet Fak Derg.

[bib1.bib5] Al-Salhie KCK, Al-Hummod SKM, Abbas RJ (2017). The effect of using different levels of pomegranate (*Punica granatum*) peel powder on productive and physiological performance of Japanese quail (*Coturnix coturnix japonica*). Livest Res Rural Dev.

[bib1.bib6] Ávila-Reyes JA, Almaraz-Abarca N, Chaidez Ayala AI, Ramírez-Noya D, Delgado Alvarado EA, Torres-Ricario R, Alanís Bañuelos RE (2018). Foliar phenolic compounds of ten wild species of Verbenacea as antioxidants and specific chemomarkers. Brazilian Journal of Biology.

[bib1.bib7] Aviram M, Dornfeld L, Rosenblat M, Volkova N, Kaplan M, Coleman R, Hayek T, Presser D, Fuhrman B (2000). Pomegranate juice consumption reduces oxidative stress, atherogenic modifications to LDL, and platelet aggregation: studies in humans and in atherosclerotic apolipoprotein E–deficient mice. Am J Clin Nutr.

[bib1.bib8] Bagri P, Ali M, Aeri V, Bhowmik M, Sultana S (2009). Antidiabetic effect of Punica granatum flowers: Effect on hyperlipidemia, pancreatic cells lipid peroxidation, and antioxidant enzymes in experimental diabetes. Food Chem Toxicol.

[bib1.bib9] Ben-Simhon Z, Judeinstein S, Trainin T, Harel-Beja R, Bar-Ya'akov I, Borochov-Neori H, Holland D (2015). A “White” anthocyanin-less pomegranate (Punica granatum) caused by an insertion in the coding region of the leucoanthocyanidin dioxygenase (LDOX; ANS) gene. PloS One.

[bib1.bib10] Bligh EG, Dyer WJ (1959). A rapid method of total lipid extraction and purification. Can J Biochem Physiol.

[bib1.bib11] Bölükbaşı ŞC, Dumlu B, Yağanoğlu AM (2023). Improved biological value of eggs due to the addition of pomegranate seed oil to laying-hen diets. Arch Anim Breed.

[bib1.bib12] Boussaa F, Zaouay F, Burlo-Carbonell F, Noguera-Artiaga L, Carbonell-Barrachina A, Melgarejo P, Hernandez F, Mars M (2020). Growing location affects physical properties,bioactive compounds, and antioxidant activity of pomegranate fruit (Punica granatum L. var. Gabsi). Int J Fruit Sci.

[bib1.bib13] Brand-Williams W, Cuvelier M, Berset C (1995). Use of free radical method to evaluate antioxidant activity. Food Science and Technology.

[bib1.bib14] Brenes A, Viveros A, Goñi I, Centeno C, Sayago-Ayerdi SG, Arija I, Saura-Calixto F (2008). Effect of grape pomace concentrate and vitamin E on digestibility of polyphenols and antioxidant activity in chickens. Poult Sci.

[bib1.bib15] Caner C, Yüceer M (2015). Maintaining functional properties of shell eggs by ultrasound treatment. J Sci Food Agric.

[bib1.bib16] Çetingül S, Iqbal A, Bayram I, Gültepe EE, Uyarlar C, Özçınar Ü (2019). Effect of pomegranate molasses on egg quality traits during different storage times in laying hens. Kocatepe Vet J.

[bib1.bib17] Eid Y, Kirrella AA, Tolba A, El-Deeb M, Sayed S, El-Sawy HB, Shukry M, Dawood MAO (2021). Dietary pomegranate by-product alleviated the oxidative stress induced by dexamethasone in laying hens in the pre-peak period. Animals.

[bib1.bib18] El-Hadary AE, Ramadan MF (2019). Phenolic profiles, antihyperglycemic, antihyperlipidemic, and antioxidant properties of pomegranate (Punica granatum) peel extract. J Food Biochem.

[bib1.bib19] El-Rashedy AH, Belal SK, Osman HE-D, Shehab GM (2011). Protective role of pomegranate on fatty liver in obesity: An experimental chemical and histopathological study. Taif Univ.

[bib1.bib20] Emami A, Nasri MF, Ganjkhanlou M, Rashidi L, Zali A (2015). Dietary pomegranate seed pulp increases conjugated linoleic and linolenic acids in muscle and adipose tissues of kid. Anim Feed Sci Technol.

[bib1.bib21] Fiesel A, Gessner DK, Most E, Eder K (2014). Effects of dietary polyphenol-rich plant products from grape or hop on pro-inflammatory gene expression in the intestine, nutrient digestibility and faecal microbiota of weaned pigs. BMC Vet Res.

[bib1.bib22] Ghahtan N, Kohanmoo MA, Habibi H (2019). Evaluation of dietary medicinal plants and algae in laying Japanese quails. J World's Poult Res.

[bib1.bib23] Gil MI, Tomas-Barberan FA, Hess-Pierce B, Holcroft DM, Kader AA (2000). Antioxidant activity of pomegranate juice and its relationship with phenolic composition and processing. J Agric Food Chem.

[bib1.bib24] Goñi I, Brenes A, Centeno C, Viveros A, Saura-Calixto F, Rebolé A, Arija I, Estévez R (2007). Effect of dietary grape pomace and vitamin E on growth performance, nutrient digestibility, and susceptibility to meat lipid oxidation in chickens. Poult Sci.

[bib1.bib25] Goth L (1991). A simple method for determi-nation of serumcatalase ac tivity and revision of reference range. Clin Chim Ac.

[bib1.bib26] Hernandez JM, Beardsworth P, Weber G (2005). Egg quality – meeting consumer expectations. Int Poult Prod.

[bib1.bib27] Hosseini-Vashan SJ, Ghaznavi T (2018). The performance and egg quality parameters effect of pomegranate pulp on laying hens in peak production. Iran J Anim Sci Res.

[bib1.bib28] Howell AB, D'Souza DH (2013). The pomegranate: Effects on bacteria and viruses that influence human health. Evid Based Complement Altern Med.

[bib1.bib29] Huang TH, Peng G, Kota BP, Li GQ, Yamahara J, Roufogalis BD, Li Y (2005). Pomegranate flower improves cardiac lipid metabolism in a diabetic rat model: Role of lowering circulating lipids. Br J Pharmacol.

[bib1.bib30] Huang TH, Yang Q, Harada M, Li GQ, Yamahara J, Roufogalis BD, Li Y (2005). Pomegranate flower extract diminishes cardiac fibrosis in Zucker diabetic fatty rats: Modulation of cardiac endothelin 1 and nuclear factor kappaB pathways. J Cardiovasc Pharmacol.

[bib1.bib31] Ishikawa S, Murakami H, Yamazaki M, Takemasa M (1999). Effect of carrot leaf Fsupplementation on egg yolk 
β
-carotene content and egg quality. Jpn Poult Sci.

[bib1.bib32] Ivy CA, Nesheim MC (1973). Factors influencing the liver fat content of laying hens. Poult Sci.

[bib1.bib33] Jacob JP, Milles RD, Mather FB (2000). Egg quality. Univ Fla Ext Inst Food Agric Sci.

[bib1.bib34] Jiménez-Moreno E, González-Alvarado JM, González-Serrano A, Lázaro R, Mateos GG (2009). Effect of dietary fiber and fat on performance and digestive traits of broilers from one to twenty-one days of age. Poult Sci.

[bib1.bib35] Jensen LS, Schumaier GW, Latshaw JD (1970). “Extra caloric” effect of dietary fat for developing turkeys as influenced by calorie-protein ratio. Poult Sci.

[bib1.bib36] Jurenka JS (2008). Therapeutic applications of pomegranate (Punica granatum L.): A review. Altern Med Rev.

[bib1.bib37] Karpe F, Dickmann JR, Frayn KN (2011). Fatty acids, obesity, and insulin resistance: Time for a reevaluation. Diabetes.

[bib1.bib38] Kostogrys RB, Filipiak-Florkiewicz A, Dereń K, Drahun A, Czyżyńska-Cichoń I, Cieślik E, Szymczyk B, Franczyk-Żarów M (2017). Effect of dietary pomegranate seed oil on laying hen performance and physicochemical properties of eggs. Food Chem.

[bib1.bib39] Lauridsen C (2019). From oxidative stress to inflammation: redox balance and immune system. Poult Sci.

[bib1.bib40] Li Y, Guo C, Yang J, Wei J, Xu J, Cheng S (2006). Evaluation of antioxidant properties of pomegranate peel extract in comparison with pomegranate pulp extract. Food Chem.

[bib1.bib41] Liu J, Han L, Zhu L, Yu Y (2016). Free fatty acids, not triglycerides, are associated with non-alcoholic liver injury progression in high-fat diet-induced obese rats. Lipids Health Dis.

[bib1.bib42] Long J, Guo Y, Yang J, Henning SM, Lee RP, Rasmussen A, Li Z (2019). Bioavailability and bioactivity of free ellagic acid compared to pomegranate juice. Food Funct.

[bib1.bib43] Lorzadeh E, Heidary Z, Mohammadi M, Nadjarzadeh A, Ramezani-Jolfaie N, Salehi-Abargouei A (2022). Does pomegranate consumption improve oxidative stress? A Systematic review and meta-analysis of randomized controlled clinical trials. Clinical Nutrition ESPEN.

[bib1.bib44] Lowry OH, Rose Brough NJ, Farr AL, Randall VJ (1951). Protein Measurement with the Folin Phenol. J Biol Chem.

[bib1.bib45] Manach C, Scalbert A, Morand C, Remesy C, Jimenez L (2004). Polyphenols: Food sources and bioavailability. Am J Clin Nutr.

[bib1.bib46] Mateos G, Jiménez-Moreno E, Serrano M, Lázaro R (2012). Poultry response to high levels of dietary fiber sources varying in physical and chemical characteristics. J Appl Poult Res.

[bib1.bib47] Matkovics B, Szabo L, Varga IS (1988). Determination of enzyme activities in lipid peroxidation and glutathione pathways. Lab Diagnoszt.

[bib1.bib48] Melgarejo-Sánchez P, Núñez-Gómez D, Martínez-Nicolas JJ, Hernandez F, Legua P, Melgarejo P (2021). Pomegranate variety and pomegranate plant part,relevance from bioactive point of view: A review. Bioresour Bioprocess.

[bib1.bib49] Moga OG, Dimienescu AB, Balan L, Dima SI, Toma NF, Bîgiu A, Blidaru A (2021). Pharmacological and therapeutic properties of Punica granatum phytochemicals: Possible roles in breast cancer. Molecules.

[bib1.bib50] Naz S, Siddiqi R, Ahmad S, Rasool SA, Sayeed SA (2007). Antibacterial activity directed isolation of compounds from Punica granatum. J Food Sci.

[bib1.bib51] Noda Y, Kaneyuka T, Mori A, Packer L (2002). Antioxidant activities of pomegranate fruit extract and its anthocyanidins: Delphinidin, cyanidin, and pelargonidin. J Agric Food Chem.

[bib1.bib52] Pagare S, Bhatia M, Tripathi N, Pagare S, Bansal Y (2015). Secondary metabolites of plants and their role: Overview. Curr Trends Biotechnol Pharm.

[bib1.bib53] Rajani J, Torshizi MK, Rahimi S (2011). Control of ascites mortality and improved performance and meat shelf-life in broilers using feed adjuncts with presumed antioxidant activity. Anim Feed Sci Technol.

[bib1.bib54] Rana TS, Narzary D, Ranade SA, Chandra R (2010). Pomegranate.

[bib1.bib55] Saha SS, Ghosh M (2009). Comparative study of antioxidant activity of 
α
-eleostearic acid and punicic acid against oxidative stress generated by sodium arsenite. Food Chem Toxicol.

[bib1.bib56] Saki AA, Rabet M, Zamani P, Yousefi A (2014). The effects of different levels of pomegranate seed pulp with multi-enzyme on performance, egg quality, and serum antioxidant in laying hens. Iran J Appl Anim Sci.

[bib1.bib57] Saki AA, Shamsollah T, Ashoori A (2019). Egg iron enrichment in response to various levels of pomegranate by-product in laying hen diet. Iran J Appl Anim Sci.

[bib1.bib58] Salim A, Deiana P, Fancello F, Molinu MG, Santona M, Zara S (2023). Antimicrobial and antibiofilm activities of pomegranate peel phenolic compounds: Varietal screening through a multivariate approach. J Bioresour Bioprod.

[bib1.bib59] Sarica M, Erensayin C (2004). Poultry products. https://pmc.ncbi.nlm.nih.gov/articles/PMC7936192/pdf/main.pdf#page=9.44.

[bib1.bib60] Sharma MK, Dinh T, Adhikari PA (2020). Production performance, egg quality, and small intestine histomorphology of the laying hens supplemented with phytogenic feed additive. Journal of Applied Poultry Research.

[bib1.bib61] IBM Corp. (2015). IBM SPSS Statistics for Windows, Version 23.0 (2015).

[bib1.bib62] Sun Y, Oberley LW, Li YA (1988). Simple method for clinical assay of superoxide dismutase. Clin Chem.

[bib1.bib63] Surai PF (2014). Polyphenol compounds in the chicken/animal diet: from the past to the future. J Anim Physiol Anim Nutr.

[bib1.bib64] Surai PF, Kochish II, Fisinin VI, Kidd MT (2019). Antioxidant defence systems and oxidative stress in poultry biology: An update. Antioxidants.

[bib1.bib65] Tietze F (1969). Enzymic method for quantitative determination of nanogram amounts of total and oxidized glutathione, applica tions to mammalian blood and other tissues. Anal Biochem.

[bib1.bib66] USDA (2000). Egg grading manual USDA AA grade. https://www.ams.usda.gov/sites/default/files/EggGradingManual.pdf.

[bib1.bib67] Wells RG (1967). Egg shell strength. Br Poult Sci.

[bib1.bib68] Xu KZ, Zhu C, Kim MS, Yamahara J, Li Y (2009). Pomegranate flower ameliorates fatty liver in an animal model of type 2 diabetes and obesity. J Ethnopharmacol.

[bib1.bib69] Yılmaz B, Usta C (2010). Nar'ın (Punica granatum) terapötik etkileri. Turk Aile Hekim Derg.

[bib1.bib70] Yoshioka T, Kawada K, Shimada T, Mori M (1979). Lipid per oxidation in maternal and cord blood and protective mechanism against activatedoxygen toxicity in the blood. Am J Obstet Gy necol.

